# Warm winters are associated to more intense West Nile virus circulation in southern Spain

**DOI:** 10.1080/22221751.2024.2348510

**Published:** 2024-04-30

**Authors:** Sergio Magallanes, Francisco Llorente, María José Ruiz-López, Josué Martínez-de la Puente, Martina Ferraguti, Rafael Gutiérrez-López, Ramón Soriguer, Pilar Aguilera-Sepúlveda, Raúl Fernández-Delgado, Miguel Ángel Jímenez-Clavero, Jordi Figuerola

**Affiliations:** a Department of Conservation Biology and Global Change, Estación Biológica de Doñana (EBD), CSIC, Seville, Spain; b CIBER of Epidemiology and Public Health (CIBERESP), Madrid, Spain; c Centro de Investigación en Sanidad Animal (CISA-INIA), CSIC, Valdeolmos, Spain; d Department of Parasitology, University of Granada, Granada, Spain; e Centro Nacional de Microbiología, Instituto de Salud Carlos III, Majadahonda, Madrid, Spain; f CIBER of Infectious Diseases (CIBERINFEC), Madrid, Spain

**Keywords:** Arboviruses, birds, emerging infectious diseases, long-term surveillance, one health

## Abstract

West Nile virus (WNV) is the most widely distributed mosquito-borne flavivirus in the world. This flavivirus can infect humans causing in some cases a fatal neurological disease and birds are the main reservoir hosts. WNV is endemic in Spain, and human cases have been reported since 2004. Although different studies analyse how climatic conditions can affect the dynamics of WNV infection, very few use long-term datasets. Between 2003 and 2020 a total of 2,724 serum samples from 1,707 common coots (*Fulica atra*) were analysed for the presence of WNV-specific antibodies. Mean (SD) annual seroprevalence was 24.67% (0.28) but showed high year-to-year variations ranging from 5.06% (0.17) to 68.89% (0.29). Significant positive correlations (*p* < 0.01) were observed between seroprevalence and maximum winter temperature and mean spring temperature. The unprecedented WNV outbreak in humans in the south of Spain in 2020 was preceded by a prolonged period of escalating WNV local circulation. Given current global and local climatic trends, WNV circulation is expected to increase in the next decades. This underscores the necessity of implementing One Health approaches to reduce the risk of future WNV outbreaks in humans. Our results suggest that higher winter and spring temperatures may be used as an early warning signal of more intense WNV circulation among wildlife in Spain, and consequently highlight the need of more intense vector control and surveillance in human inhabited areas.

## Introduction

West Nile virus (WNV) belongs to the *Orthoflavivirus* genus (family *Flaviviridae*) and is the most widely distributed mosquito-borne flavivirus in the world [1]. Birds are the main competent hosts, with different mosquitoes of the genus *Culex* acting as the key vectors [2–4]. WNV is a generalist pathogen able to infect hundreds of bird species, among other vertebrates, although the contribution of different bird species to viral amplification, and the occurrence of outbreaks is highly variable [5–7]. Previous studies have identified the common coot (*Fulica atra*) as a relevant species for WNV surveillance in the Palearctic, because this species has a high WNV seroprevalence [8, 9].

Mammals, including humans, are considered dead-end hosts of WNV because they reach low viremias during infection that are not sufficient to infect mosquitoes feeding on their blood [10]. In humans, WNV infections most often course asymptomatically, while 20% may show disease signs ranging from mild fever to more severe illnesses, such as acute encephalitis, poliomyelitis, meningitis, or hepatitis, with a fatality rate of less than 1% [11]. WNV is considered a re-emerging pathogen in Europe due to its increasing but highly variable incidence [12].

In Spain, primarily in the southern area, WNV circulation is known since 2003 as confirmed by seroconversion of resident birds and horses [8, 13–15], and the detection of the virus in mosquitoes and birds [16–18]. Human cases were sporadically detected in the region (1 clinical case in 2004, 2 in 2010, and 3 in 2016) until 2020, when an unprecedented outbreak caused 77 clinical cases and eight fatalities [19, 20].

Climate factors, such as temperature and precipitation, have been associated with the increase in incidence of WNV in Europe as reviewed by Brugueras et al. [21]. However, long-term longitudinal studies, which are still scarce [3, 22], are necessary to clarify the relationships between climate and virus incidence in humans, avian reservoirs, and mosquitoes [3].

This study focused on a population of common coots known to have been exposed to WNV infection, at least, since 2003. The study aimed to investigate the impact of climatic conditions on WNV exposure within this population. For that, we assessed WNV seroprevalence annually over an 18 years period (2003–2020) and analysed the relationship between WNV prevalence and local climatic parameters.

## Materials and methods

Between 2003 and 2020 we collected 2,724 blood samples from 1,707 common coots (range: 1–13 samples/individual) at the *Cañada de los Pájaros* (37°14′N 6°07′W) in southwestern Spain. The study area has a Mediterranean climate characterized by large annual fluctuations in precipitation that generate important variations in the breeding success of the study species and its abundance in the sampling area, in addition to the different projects that finance this long term study also favour the differences found in the size of the sampling between years. Although birds were captured monthly using a walk-in trap, more than 60% of captures were done between October and December. Each individual was marked with a numbered aluminium ring and released immediately after handling. We determined bird age according to Baker [23] and took up to 1 ml of blood from the femoral vein using sterile syringes, never exceeding 1% of the body mass. The blood was collected in 2.5 ml TapVal™ plastic (polypropylene) tubes, that are kept in portable icebox during the collection process, and stored to 4°C until centrifugation within the next 24 h.

Temperature and precipitation records for the study period were obtained from the closest meteorological station, located at 1.5 km (https://www.juntadeandalucia.es/agriculturaypesca/ifapa/riaweb/web/estaciones?provincia_filter=41). For analysis, we selected climatic variables based on prior studies that have investigated the correlation between climate and WNV circulation [3, 24–26] (Supplementary Table 1).

### Antibody detection assays

WNV antibodies were detected in serum samples using two approaches. Neutralizing antibody (NtAb) titres were monitored during the whole 18-year period of study, using a previously described virus-neutralization test (VNT) detailed in Llorente et al. [27]. In 2005, when a monoclonal antibody-based blocking ELISA became available commercially (INgezim West Nile Compac, Ingenasa, Spain) [28], it was introduced in the diagnostic scheme and used as the first screening method. When samples were positive or doubtful in the ELISA assay, a confirmatory VNT was carried out. Specificity was assessed by comparing NT Ab titres measured in parallel against WNV and another flavivirus, Usutu virus (USUV), also circulating in the area [15]. VNT titres were attributed to serum dilutions of 1:10 or higher, producing complete neutralization of the cytopathic effect observed in the cell monolayer. Specificity was attributed for each individual sample to the virus that it neutralized *in vitro* at titres at least four times higher than the observed for the other virus, following Calisher et al. [29]. Samples not reaching this threshold were considered “positive for an undetermined flavivirus”. This undetermined flavivirus samples may be due to low levels of antibodies due to past infections by WNV, individuals that have been simultaneously or serially been infected by WNV and Usutu, or individuals infected by an unknown but antigenically related flavivirus circulating in the area. In this study, we only considered as positive those samples confirmed positive for WNV, while samples positive for undetermined flaviviruses were considered as negatives in the analyses. However, the results of our analyses did not change qualitatively when repeated considering undetermined flavivirus samples as positive for WNV.

### Statistical analysis

We fitted two different generalized linear mixed-effects models (GLMM) to our common coots serology dataset. The first one included all the samples from the trapped individuals to determine the impact of age and season on WNV seroprevalence. The second one was restricted to samples from less than one year old birds to analyse the relationship between climate variables and WNV exposure. We fitted a generalized linear mixed-effects model (GLMM) with a “logit” link function and a binomial distribution to assess the impact of age class (juvenile: <1 year; adult: >1 year), and atmospheric season (spring, summer, autumn, and winter) on the serological status of common coots (whether they tested positive or negative for the presence of antibodies against WNV). Seasons were defined as autumn (22 of September to 21 of December), winter (20 of December to 20 of march), spring (21 of march to 21 of June) and summer (22 of June to 21 of September). The year of sampling and the individual common coot identity (ID) were included as random factors to account for the lack of independence among samples collected within the same year or from the same individual. Statistical significance for the categorical factors and post-hoc tests were assessed using ANOVA type III chi-square tests and contrast of Least-Squares Means (LSMeans), respectively. To control the effects of the variables included in the statistical models, seroprevalence was estimated from the back-transformed LSMeans estimates, now referred to as Estimated Marginal Means (EMMs) (see Supplementary Table 2) and we used delta method to calculate the back-transformed standard deviations.

We fitted a second GLMM model to investigate the relationship between climate variables and WNV seroconversion. In this case, we considered only one sample per individual, which was collected during their first year of life, to determine that the serological status reflects WNV infection on the sampling year. We included samples collected between October and December from 2003 to 2020, except for 2009 and 2011, when no juveniles were captured. The response variable in this model was the serological status confirmed by VNT, while the climate variables were the independent variables (Supplementary Table 1). We employed a forward stepwise selection procedure according to AIC to construct the most appropriate regression model, including significant climate predictor variables related to the response variable. Due to the lower specificity of the ELISA test for USUV as compared to WNV, and that USUV antibodies were only detected in two juvenile and one adult coots in 2020, all the analyses were confined to WNV-positive cases.

The collinearity between independent variables included in each GLMM was tested with the variance inflation factor (VIF) [30]. VIF values were lower than 2.9 in all the cases. Overdispersion did not significantly deviate from one, as estimated by the Pearson statistic. Temporal autocorrelation was also evaluated using the Breusch–Godfrey test (LM test = 1.784, *p* = 0.182), indicating no evidence of serial correlation. All statistical analyses and figures were done in R (v. 4.2.2; The R Foundation for Statistical Computing Platform 2020).

## Results

**Figure 1.**
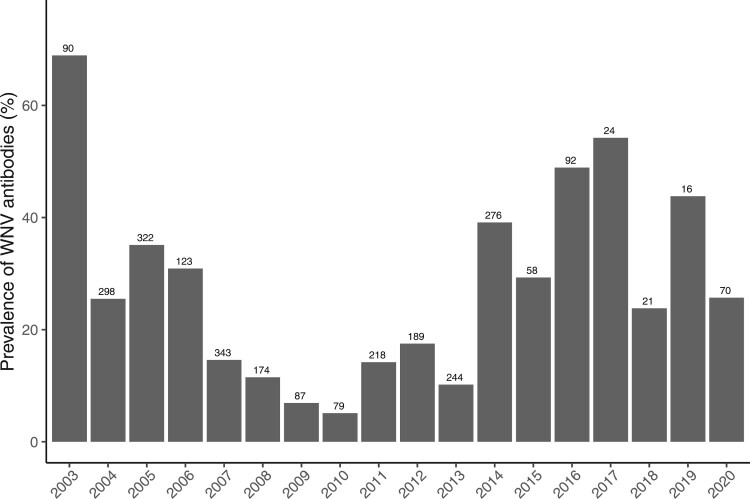
Mean annual prevalence of WNV antibodies in common coots sampled between 2003 and 2020. Bars indicate seroprevalence per year while the number of individuals analysed each year is indicated in the numbers above bars.

A total of 833 samples collected between 2003 and 2006 were only tested using VNT, out of which 289 were positive for WNV antibodies. From 2007 to 2020, 1891 samples were tested using ELISA, with 148 samples being identified as doubtful and 891 as positive for WNV antibodies. The positive and doubtful samples underwent VNT analysis, resulting in 383 positive samples for WNV. Additionally, other 286 samples positive by VNT were categorized as positive for undetermined flavivirus, and 3 samples from 2020 were positive for USUV. The prevalence of WNV antibodies varied across the years, showing a mean (SD) seroprevalence of 24.67% (0.28), ranging from 5.06% to 68.89% (Figure 1).

**Figure 2.**
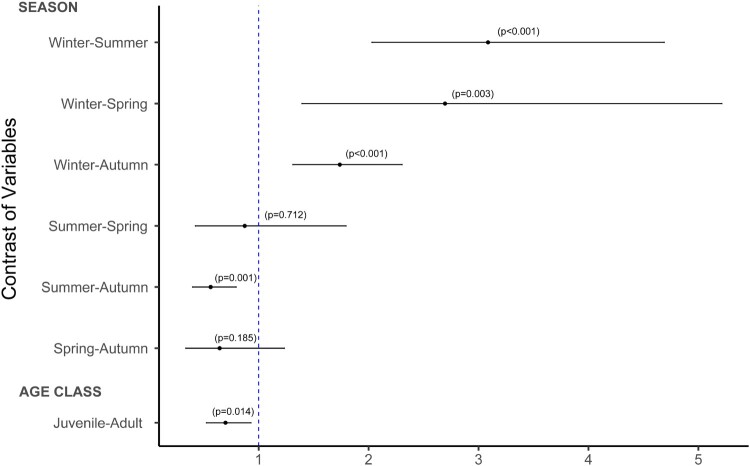
Odds ratios plot with 95% confidence intervals showing the ratio of odds between seasons (winter, spring, summer and autumn) and between age classes (juveniles and adults). The blue dotted line represents the statistical probability (p) that the odds ratio equals 1.

The prevalence of WNV antibodies was higher in adult than in juvenile coots (*χ*^2^ = 6.04, d.*f*. = 1, *p* = 0.01); (Figure 2) and varied across seasons (*χ*^2^ = 32.66, d.*f*. = 3, *p* < 0.01), with significantly higher seroprevalences in winter and autumn than in summer, and in winter than in spring (Figure 2, Supplementary Table 2 and 3).

**Figure 3.**
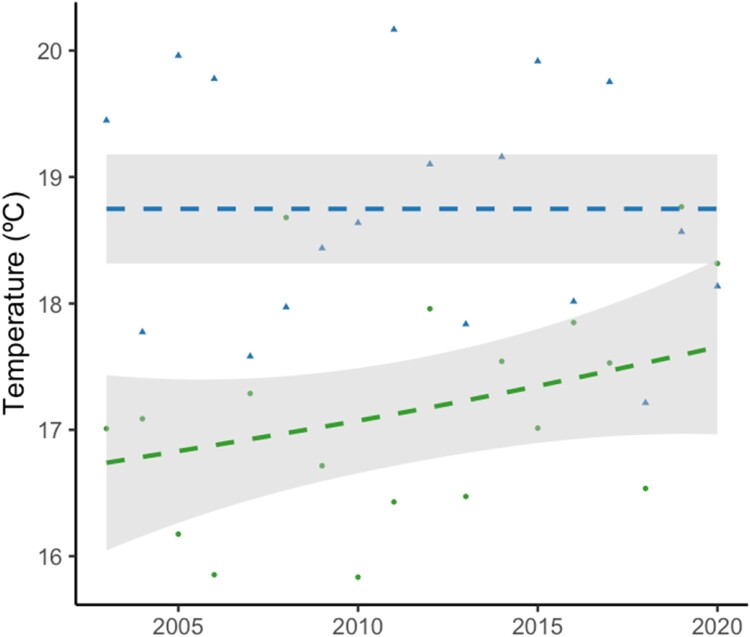
Scatter plot showing the relationship between mean maximum temperature in winter (green), mean temperature in spring (blue) during sampling period in year. The grey shaded area represents the smoothed trend with a span of 0.5.

**Figure 4.**
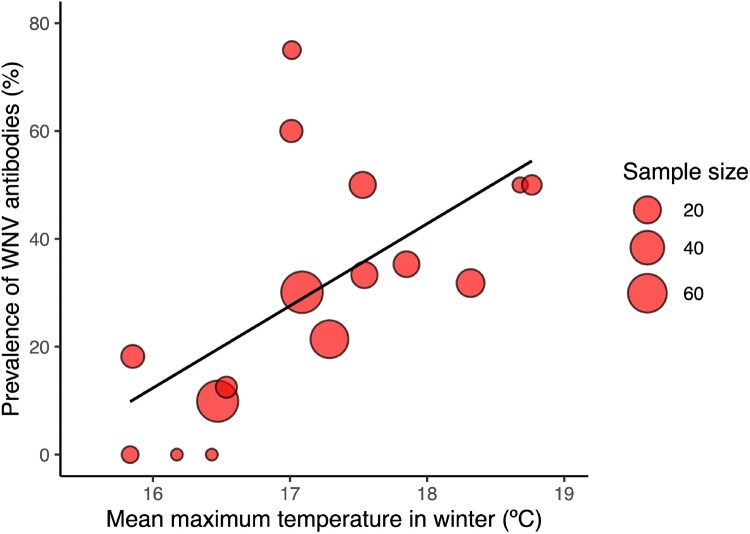
Bubble plot showing the relationship between the prevalence of WNV antibodies (%) and mean maximum temperature in winter. The size of the cycles is proportional to the number of juvenile common coots tested.

The seroprevalence in young common coots sampled between October and December of each year was of 26.47% (0.28). The analysis of this dataset show that the seroprevalence of WNV antibodies was positively associated with the mean maximum temperature in winter (Figure 3) (Estimate ± S.E. =  0.684 ± 0.21, z = 3.18, *p* < 0.01) (Figure 4) and mean temperature in spring (Figure 3) (Estimate ± S.E. =  0.512 ± 0.17, z = 2.90, *p* < 0.01). The addition of the remaining variables did not significantly improve the model fit (Supplementary Table 4).

From 340 individuals sampled in consecutive years, 122 tested positive for WNV antibodies, including 70 that exhibited seroconversions, i.e. transitioned from negative when tested on the first year to positive when tested the following year, and 41 coots seronegativized, i.e. shifted from positive to negative between tests in consecutive years.

## Discussion

### Long-term analysis of WNV antibodies in common coots

Although WNV circulation has been known since 2003 [8] in Spain, cases in humans were sporadic until the outbreak in 2020 [19]. Our study confirms that WNV can circulate silently for years. The circulation of WNV in southern Spain has been continuous during the last two decades, with a very high mean prevalence of WNV antibodies in common coots (32.74%) that varies along the study period. The evolution of WNV seroprevalence in the study population reveals three periods: (i) from 2003 to 2006, with a high or very high (2003) seroprevalences, followed by (ii) a second period (2007–2013) when seroprevalence declined to values lower than the mean value of the total period (reaching a minimum in 2010) and (iii) a third period starting in 2013. Since 2013, the seroprevalences increased significantly above the mean, with several years of high prevalence remarkably preceding the 2020 human outbreak. In addition, WNV seroprevalence in common coots was appreciably higher than in horses ranging in the same area [25]. Nevertheless, in both populations the changes in WNV seroprevalence showed a parallel pattern, alternating high and low seroprevalence values in approximately the same periods. When analysed seasonally, the highest seroprevalences were observed in winter and autumn, suggesting that they arise as a result of seroconversions occurring during the summer-autumn months when mosquito activity is higher [8]. Similar patterns were observed in other studies involving horses [25].

### WNV and USUV antibodies in common coots

WNV seroprevalence was higher in adult birds which may be due to their prolonged exposure to infected mosquito bites over time. Such dynamics have been previously observed [33, 34] and agree with the notion that cumulative exposure increases seroprevalence. However, in agreement with previous longitudinal observations [8, 13], our results show that antibodies are not detectable lifelong, because slightly more than 12% individuals seroreverted years after exposure. Thus, the seroprevalence in adult birds reflects the final equilibrium between seroconversions due to new and past exposures to the virus and antibody decay. Hence, naïve and seroreverted birds cannot be distinguished using currently available techniques. Seroconversions are expected to occur mainly in juveniles, i.e. during the first summer after hatching, thus the analyses of antibody prevalence in the juvenile subset are important since they may reflect the intensity of WNV circulation in a given year.

The seroprevalence of USUV antibodies found in coots was lower than found in a nearby (30 km) feral horse population. While in horses USUV-positive individuals were detected between 2011 and 2018, with a mean seroprevalence of 10.37% [25], only three USUV-positive coots were detected in the present study, all in 2020. This finding could be explained based on hypothetical differences in susceptibility to USUV infection between horses and common coots, or more probably, different exposure due to ecological factors such as those conditioning the abundance and diversity of the different mosquito species [15, 35]. However, we cannot discard that the lower prevalence of USUV-specific antibodies in coots was an artefact due to the lower percentage of sera scored as of unidentified flavivirus in horses than in coots (2.8% vs 15.11%).

### Implication of climate in WNV seroprevalence

Climate has the potential to strongly impact the transmission of vector-borne pathogens, primarily due to its effects on the development of mosquito populations, as influenced by factors such as rainfall and temperature [36–38]. In addition, the temperature can also significantly influence virus development and transmission by the vectors themselves [39, 40], affecting WNV replication, reducing the extrinsic incubation period, and increasing virus load in vectors [41]. These effects may potentially explain the positive association between the WNV seroprevalence of the young common coots and the mean maximum temperature in winter and mean temperature in spring. Maximum temperatures during the winter may be related to higher vector survival during the winter and the start of early reproduction, two factors that may facilitate virus survival over winter and early replication. The effects of temperature extend to multiple aspects, including the length of extrinsic incubation period, seasonal patterns of mosquito host populations, and geographical variations in human case incidence [36].

Several studies have consistently linked higher temperatures during the spring and summer months to an increased incidence of WNV disease cases in humans across Europe [42, 43]. Moreover, elevated temperatures prompt heightened vector population growth rates, reduced intervals between blood meals, accelerate the incubation period from infection to infectiousness in mosquitoes, enhanced the virus evolution rate, and increased viral transmission efficiency to birds [36, 41].

Winter and spring temperatures are expected to rise in southern Spain over the next decades according to the regionalization for Spain of the Hadley Centre Global Environmental Model version 2 with Earth System components (HadGEM2-ES); [31, 32] provided by AEMET and available from https://escenarios.adaptecca.es/. Because these variables are positively associated to seroprevalence in our study, we can expect an increase in the prevalence of WNV in the next years. However, this situation may not follow a linear trajectory, as other factors could affect virus replication and circulation. For instance, the effect of a high seroprevalence of antibodies in the avian hosts on the rate of virus circulation in the vectors should be considered as well as the effects of climate change in vector populations in the area [44]. In addition, bird communities may also be affected by climate change, specially affected by changes in precipitation that may result in more frequent and intense droughts that may negatively affect birds populations in the area [45, 46], and increase their dispersal to other wetlands with better hydrologic conditions [47, 48]. This effect may be observed in the seroprevalence data detected in this study in 2003 when a seroprevalence of 75% was observed, followed by a drastic decrease in 2004, with the seroprevalence being threefold lower. These results indicate that WNV will continue to pose a significant threat to both animal and public health in southern Europe.

## Conclusion

Over two decades, WNV has become endemic in southern Spain and many other areas in Europe, with a strongly variable incidence across the years. The results reveal a noteworthy increase in seroprevalence in birds during the last years, indicating a rising intensity of WNV circulation in southern Spain. The positive relationship between temperatures in the preceding winter and spring and WNV seroprevalence in common coots sampled during the autumn suggests potential impacts on vector survival, behaviour, viral replication rates, and transmission efficiency, which favour increased WNV activity. Due to the apparent importance of high winter temperatures for the high levels of WNV circulation, a deeper understanding of the factors involved in virus overwintering and early replication is necessary. Such knowledge is crucial for implementing effective control and prevention measures in winter to mitigate the potential risks posed by WNV to both animal and human populations. As the prevalence of vector-borne diseases like WNV continues to be a global concern, ongoing research under the One Health paradigm is imperative to inform public health surveillance, intervention, and communication strategies.

## Supplementary Material

Supplementary_Material_Hot_winters_precede_more_intense_WNV_clean
